# Rapid parallel measurements of macroautophagy and mitophagy in mammalian cells using a single fluorescent biosensor

**DOI:** 10.1038/srep12397

**Published:** 2015-07-28

**Authors:** A. Sargsyan, J. Cai, L. B. Fandino, M. E. Labasky, T. Forostyan, L. K. Colosimo, S. J. Thompson, T. E. Graham

**Affiliations:** 1 Molecular Medicine Program and Division of Endocrinology, Metabolism & Diabetes, Department of Medicine, University of Utah, Salt Lake City, UT, USA 84112; 2 Department of Biochemistry, University of Utah,Salt Lake City, UT, USA 84112; 3 Department of Oncological Sciences, University of Utah, Salt Lake City, UT, USA 84112

## Abstract

Mitochondrial dysfunction is implicated in many human diseases and occurs in normal aging. Mitochondrial health is maintained through organelle biogenesis and repair or turnover of existing mitochondria. Mitochondrial turnover is principally mediated by mitophagy, the trafficking of damaged mitochondria to lysosomes via macroautophagy (autophagy). Mitophagy requires autophagy, but is itself a selective process that relies on specific autophagy-targeting mechanisms, and thus can be dissociated from autophagy under certain circumstances. Therefore, it is important to assess autophagy and mitophagy together and separately. We sought to develop a robust, high-throughput, quantitative method for monitoring both processes in parallel. Here we report a flow cytometry-based assay capable of rapid parallel measurements of mitophagy and autophagy in mammalian cells using a single fluorescent protein biosensor. We demonstrate the ability of the assay to quantify Parkin-dependent selective mitophagy in CCCP-treated HeLa cells. In addition, we show the utility of the assay for measuring mitophagy in other cell lines, as well as for Parkin-independent mitophagy stimulated by deferiprone. The assay makes rapid measurements (10,000 cells per 6 seconds) and can be combined with other fluorescent indicators to monitor distinct cell populations, enabling design of high-throughput screening experiments to identify novel regulators of mitophagy in mammalian cells.

Mitochondria perform diverse cellular functions, including energy production, intermediary metabolism, and calcium and apoptosis regulation. Mitochondrial dysfunction, characterized by impaired oxidative phosphorylation and excessive production of reactive oxygen species (ROS), contributes to many human diseases^1^,^2^,^3^,^4^,^5^. Mitochondrial ROS regulate normal cellular functions and stress responses^6^; however, excessive ROS production can damage proteins, lipids, and other cellular components, including mitochondria themselves^7^, leading to further worsening of mitochondrial dysfunction. Mitochondria possess several quality control mechanisms to counteract damage and maintain functionality^8^,^9^,^10^. Important among these is mitophagy, the trafficking of intact mitochondria or mitochondrial fission products via autophagy to lysosomes where they are degraded. Mitophagy is also required for developmental processes that require mitochondrial clearance, such as destruction of paternal mitochondria in oocytes after fertilization^11^ and maturation of erythrocytes^12^, eye lens epithelium^13^, and adipocytes^14^. Perturbations in mitophagy have been implicated in heart disease^15^,^16^,^17^, neurodegeneration^18^,^19^,^20^ , metabolic syndrome, diabetes^21^,^22^ and cancer^23^,^24^. Impairment of autophagy or mitophagy drastically alters mitochondrial function and cell fate in many cell types, indicating the importance of these pathways.

Mitophagy requires intact autophagy; however, induction of autophagy alone is not sufficient for mitophagy to occur. Increasing evidence indicates that mitophagy is a highly selective process. Whether or not individual mitochondria are trafficked into the autophagy pathway depends on factors such as mitochondrial membrane potential and display of certain proteins or lipids in the mitochondrial outer membrane that act as molecular adaptors to engage mitochondria with nascent autophagasome membranes^25^,^26^,^27^,^28^,^29^. These molecular cues are capable of relaying the integrity and functional state of individual mitochondria, such that damaged or impaired mitochondria are targeted for destruction and functional mitochondria are preserved. Similar mechanisms also appear to coordinate induction of mitophagy during development in certain cell types. Therefore, when assessing potential mechanisms involved in regulating mitophagy, there is value in assessing the states of both autophagy and mitophagy, together and separately.

HeLa cervical carcinoma cells treated with carbonyl cyanide m-chlorophenylhydrazone (CCCP) are a widely studied model of selective mitophagy. CCCP uncouples the electron transport chain and causes varying degrees of mitochondrial inner membrane depolarization. In most cells, CCCP induces generalized autophagy^30^,^31^, along with mitophagy that selectively targets depolarized mitochondria for destruction^32^. Activation of the Parkin E3 ubiquitin ligase, initiated by Pink1 stabilization on depolarized mitochondria, is a proposed mechanism for selectivity in this process^33^. HeLa are particularly noteworthy as a model for Parkin-dependent selective mitophagy because they do not express endogenous Parkin, making it possible to assess the requirement for Parkin by expressing it ectopically^34^.

We sought to employ the CCCP-treated HeLa cell as a model for developing an assay capable of rapid, highly quantitative parallel measurements of autophagy and mitophagy, with the long term goal of enabling high throughput screens for regulators of mitophagy in mammalian cells. The Rosella biosensor has been used to measure mitophagy in yeast^35^,^36^. Rosella is a chimera of two tandem fluorescent proteins: pHluorin a pH-sensitive green fluorescent protein^37^, and dsRed.T3^38^, a non-pH-sensitive red fluorescent protein (Fig. 1A). Attaching Rosella to specific cellular ‘cargos’ makes it possible to monitor their separate delivery to the acidic compartments of lysosomes via autophagy. We hypothesized that Rosella-LC3 and Mito-Rosella could be used together for simultaneous measurement of autophagy and mitophagy in a single parallel assay capable of demonstrating the requirement for Parkin in selective mitophagy in CCCP-treated HeLa cells. Here we report the performance characteristics of Rosella-LC3 and Mito-Rosella biosensors in single-cell and flow cytometry (FCM)-based parallel autophagy/mitophagy assays, and describe the potential of this method for conducting rapid, high-throughput assays in human cells.

## Results

### Mito-Rosella localization and pH-dependence

**Figure 1:**
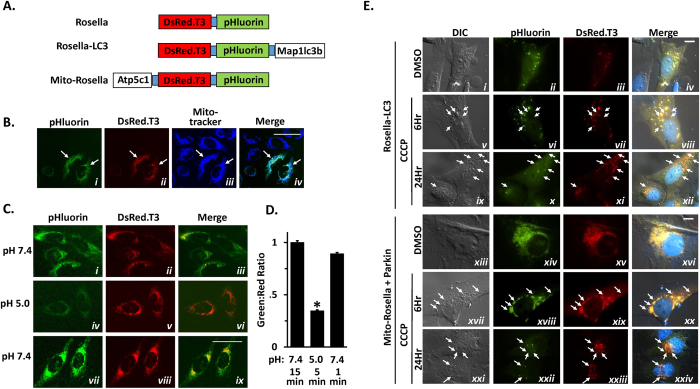
Rosella pH-responsive fluorescent biosensors. (**A**) Schematic representation of Rosella, Rosella-LC3, and Mito-Rosella fluorescent protein biosensor constructs. Rosella (top) is a chimeric protein containing the pH-stable DsRed.T3 red fluorescent protein and the pH-sensitive pHluorin green fluorescent protein. Rosella-LC3 (middle) consists of the complete open reading frame (ORF) of human LC3B (Map1lc3b) joined in-frame with the 3′ end of the Rosella ORF (coding the C-terminus) without a stop codon. Mito-Rosella (bottom) consists of the complete human ATP synthase subunit gamma (Atp5c1) ORF without a stop codon fused in-frame with the 5′ end of the Rosella ORF (coding the N-terminus). (**B**) Representative micrographs of HeLa cells transiently transfected with expression plasmids for Mito-Rosella and treated with Mitotracker Deep Red FM to image mitochondria independently of Rosella. The merged panel shows overlap of fluorescence from the green, red, and far-red channels in two cells expressing Mito-Rosella (white arrows). (**C)** Representative micrographs of green and red channel fluorescence of Mito-Rosella in cells subjected to sequential incubations in culture media adjusted to pH 7.4 (for 15 min; starting point), pH 5.0 (for 5 min), and then pH 7.4 again (for 1 min). (**D**) Quantification of green to red fluorescence ratios measured in single cells subjected to pH changes described above for (**C**). Bars represent mean ± SEM of ratios for n = 50–60 cells per condition. *p < 0.05, based on unpaired T-test. For panels B and C: scale bars indicate a length of 50 μm. (**E**) Representative widefield micrographs of HeLa cells transiently transfected with expression plasmids for Rosella-LC3 (panels i to xii) and Mito-Rosella (panels xiii to xxiv) with Parkin, treated with CCCP (10 μM) or vehicle for 6 and 24 hr. In merged images arrows represent acidified Rosella-LC3 (panels viii and xii) and acidified Mito-Rosella (panels xx and xxiv). DIC, differential interference contrast microscopy. Scale bars indicate a length of 10 μm.

Transiently transfected Mito-Rosella specifically and completely labeled mitochondria (Fig. 1B), whereas Rosella lacking a targeting sequence showed diffuse localization (Suppl. Fig. 1). Rosella green fluorescence was reduced 70% without affecting red fluorescence when culture media pH was reduced to 5.0, and recovered rapidly with return to pH 7.4. (Fig. 1C,D). Expression of Rosela-LC3 or Mito-Rosella did not alter the dynamics or extent of mitophagy and autophagy compared to control transfection, as measured by Western blot analysis of mitochondrial markers (Suppl. Fig. 2A) and endogenous LC3 lipidation (Suppl. Fig. 2B).

### CCCP-induced redistribution of Rosella-LC3 and Mito-Rosella to lysosomal compartments

Vehicle (DMSO)-treated HeLa cells expressing Rosella-LC3 exhibited dual-labeled green/red puncta throughout the cytoplasm, consistent with the characteristic appearance of autolysosomes (Fig. 1E, i–iv). In cells treated with CCCP (10 μM), red-only vesicles were present at 6 hr after treatment (Fig. 1E, v–viii) and increased in number at 24 hr after treatment (Fig. 1E, ix–xii). Vehicle-treated HeLa cells co-expressing Mito-Rosella and Parkin exhibited dual-labeled green/red mitochondria throughout the cytoplasm (Fig. 1E, xiii–xvi). Treatment with CCCP resulted in appearance of Mito-Rosella in red-only vesicles, indicative of redistribution to acidic lysosomal compartments, located in the cytoplasm at 6 hr after treatment (Fig. 1E, xvii–xx), and accumulating near the perinuclear lysosomal compartments at 24 hr after treatment (Fig. 1E, xxi–xxiv).

### Parkin-dependent selective mitophagy demonstrated by differential responses of Rosella-LC3 and mito-Rosella

**Figure 2:**
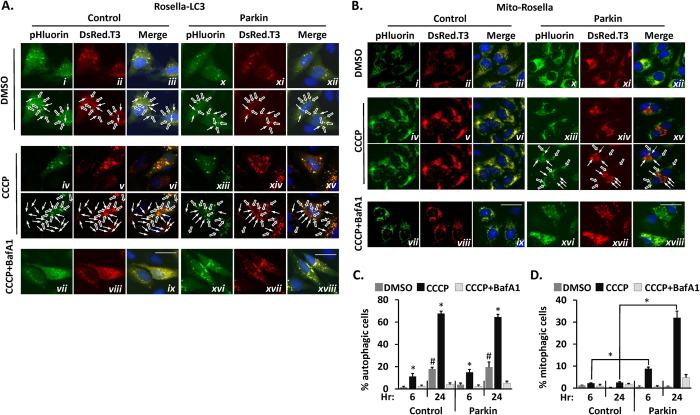
Single-cell measurements of autophagy and mitophagy based on biosensor responses to CCCP treatment. (**A**) Representative micrographs of HeLa cells transiently co-transfected with Rosella-LC3 expression plasmid plus Parkin expression plasmid or ‘empty’ plasmid (Control). Cells were treated for 24 hr with: Vehicle alone (DMSO), CCCP (10 μM), or CCCP (10 μM) + lysosomal inhibitor Bafilomycin-A1 (BafA1, 100 nM). CCCP-treated cells displayed accumulation of primarily red Rosella-LC3 fluorescence near the central lysosomal compartments (solid arrows in identical, labeled panels below panels vi and xv), while vehicle treated cells primarily display co-localization between red and green signals (hollow arrows in identical, labeled panels below panels iii and xii). Co-treatment with BafA1 (panels ix and xviii) prevented CCCP-induced relocation of Rosella-LC3 to the central lysosomal compartment, causing it to accumulate in autophagasomes within the cytoplasm (panels ix and xviii). (**B**) Representative micrographs of HeLa cells co-transfected with Mito-Rosella plus either Parkin or Control plasmids, and treated exactly as described above for (A). CCCP treatment caused an accumulation of red-only Mito-Rosella-labeled mitochondria in the central lysosomal compartments in Parkin co-transfected cells but not in control cells (solid arrows in identical, labeled panel below panel xv), and this was inhibited by BafA1 treatment (panel xviii). In contrast, DMSO-treated Parkin-expressing cells (panel xii) and control cells treated with vehicle (DMSO) or CCCP (panels iii and vi), there is a complete co-localization of green/red fluorescence. Quantification of percentage of cells exhibiting induction of (**C**) autophagy or (**D**) mitophagy, as determined by categorizing individual cells on the basis of presence/absence of visible accumulations of red biosensor fluorescence in the lysosomal compartments as in (A) and (B) above. For panels C and D: bars represent mean ± SEM of percentages of cells from n = 4 biological replicates, with >120 cells categorized per replicate. For panels A and B: scale bars indicate a length of 25 μm. For panel C: *p <  0.05 vs. DMSO treated cells at the same time-point; and ^#^*p*  <  0.05 vs. 6 hr time-point by unpaired T-test. For panel D: *p <  0.05 by unpaired T-test for the indicated comparisons between cells co-transfected with Parkin vs. Control plasmid.

To demonstrate the dependence of mitophagy on Parkin expression in CCCP-treated HeLa cells, we analyzed autophagy (*i.e.,* LC3-Rosella) and mitophagy (*i.e.,* Mito-Rosella) responses in the presence or absence of co-expressed Parkin. HeLa cells transiently transfected with Rosella-LC3 were treated with vehicle (DMSO) or CCCP for 24 hr. In vehicle-treated cells, Rosella-LC3 produced primarily dual-labeled green/red autophagic puncta (Fig. 2A, i–iii), while CCCP treatment induced large perinuclear accumulations of red-only LC3, indicating redistribution of autophagasomes to low pH lysosomal compartments (Fig. 2A, iv–vi). Treatment with Bafilomycin-A1 (BafA1), an inhibitor of the lysosomal V-ATPase that maintains lysosome pH, caused accumulation of dually labeled green/red LC3 puncta (Fig. 2A, vii–ix), indicative of autophagasomes or autolysosomes collecting under conditions of impaired lysosome acidification. Co-expressing Parkin did not alter the number, size or localization pattern of LC3-containing structures, relative to control cells under the same conditions (Fig 2A, x–xviii).

In vehicle-treated cells transfected with Mito-Rosella, mitochondria were primarily dual-labeled green/red and exhibited normal cytosolic localization (Fig. 2B, i–iii). CCCP treatment (10 μM, 24 hr) did not alter mitochondrial fluorescence or localization in control cells (Fig. 2B, iv–vi). In cells expressing Parkin, CCCP induced redistribution and accumulation of red-only mitochondria near the central lysosomal compartments (Fig. 2B, xiii–xv) in a BafA1-dependent manner (Fig. 2B, xvi–xviii). Therefore, LC3-Rosella and Mito-Rosella responses replicate prior observations that autophagy occurs in a Parkin-independent manner whereas mitophagy occurs in a selective, Parkin-dependent manner in CCCP-treated HeLa cells.

Autophagy and mitophagy were quantified in single cells by blinded scoring of Rosella-LC3 and Mito-Rosella responses, respectively. CCCP induced autophagy in 11.2 ± 2.6% of cells at 6 hr (vs. 2.4 ± 1.0% in CCCP + BafA1-treated cells, *p* < 0.05, Fig. 2C) and 67.5 ± 2.2% of cells at 24 hr (vs. 4.0 ± 1.4% in CCCP + BafA1-treated cells, *p* < 10^−8^, Fig. 2C). Interestingly, cells in vehicle-treated media exhibited increased autophagy after 24 hr (17 ± 1.4% vs. 1.4 ± 0.9% baseline, *p* < 0.001, Fig. 2C), though to a much lesser extent than CCCP treatment. Co-expression of Parkin did not alter the autophagic response in vehicle or CCCP-treated cells (*p* = NS for LC3-Rosella alone vs. LC3-Rosella co-expressed with Parkin at 6 or 24 hr time-points after treatment, Fig. 2C). In cells expressing Mito-Rosella, CCCP-induced mitophagy solely in cells co-expressing Parkin (8.8 ± 0.8% with Parkin vs. 2.2 ± 0.2% without Parkin at 6 hr, *p* < 0.001; and 32 ± 3.0% with Parkin vs. 2.5 ± 0.4% without Parkin at 24 hr, *p* < 10^−4^, Fig. 2D).

### Flow-cytometry based parallel analyses of autophagy and mitophagy using Rosella biosensors

**Figure 3:**
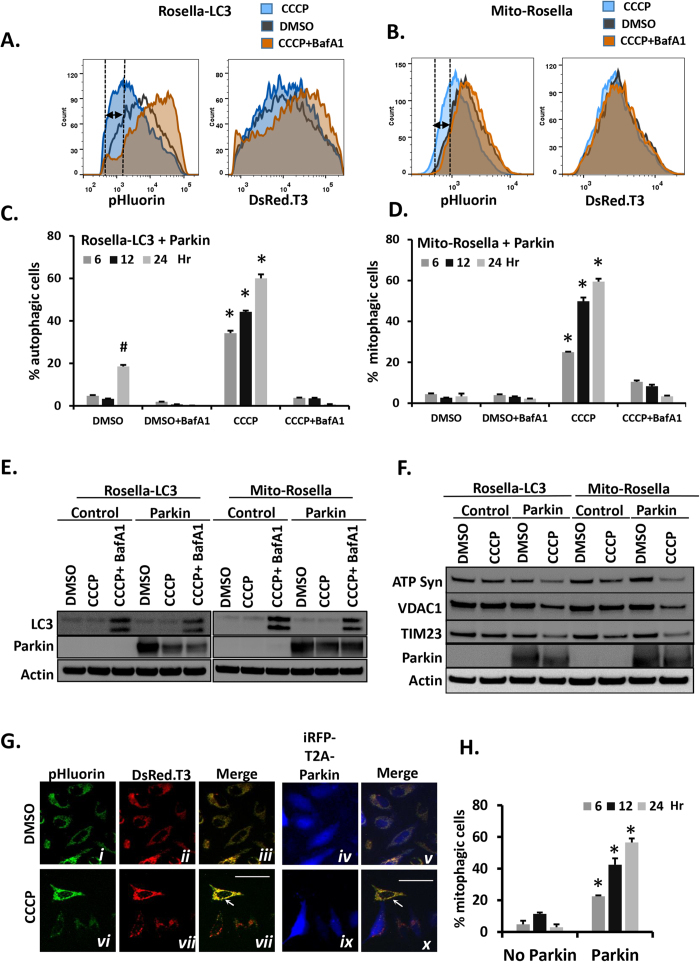
Parallel quantification of autophagy and mitophagy under the same conditions by flow cytometry (FCM) measurement of biosensor responses. Representative histograms of pHluorin/green and DsRed.T3/red fluorescence intensities of HeLa cells transiently transfected with plasmids expressing (**A**) Rosella-LC3 and Parkin, or (**B**) Mito-Rosella and Parkin, and treated for 24 hr with: DMSO (vehicle), CCCP (10 μM), or CCCP (10 μM) + Baf1A (100 nM); histograms depict intensities of n = 10,000 cells per condition. CCCP induced a significant reduction (dashed lines and arrow) in pHluorin/green fluorescence but not in DsRed.T3/red fluorescence of both Rosella-LC3 and Mito-Rosella. Quantitation of cells with (**C**) increased autophagy or (**D**) increased mitophagy based on detection of changes in green vs. red fluorescence intensities in gated FCM scattergram analysis (see also Suppl. Fig. 5A–B); bars represent means of 3 biological replicates ± SEM, N = 10,000 cells analyzed per replicate; *p < 0.001 vs. CCCP + BafA1; #*p* < 0.001 vs. DMSO at 6 time-point (see also Suppl. Fig. 5A–B). (**E**) Western blots of LC3 processing in cells co-transfected with Rosella biosensors plus Parkin or Control plasmids and treated 24 hr as indicated. (**F**) Western blots of mitochondrial markers in cells co-transfected with plasmids for each of the Rosella biosensors plus Parkin or Control plasmid. (**G**) Representative micrographs of HeLa cells stably expressing Mito-Rosella and transiently transfected with a bicistronic iRFP-T2A-Parkin expression plasmid; iRFP fluorescence was readily visualized and exhibited a diffuse localization in successfully transfected cells (panels iv and ix). As expected, DMSO did not induce mitophagy in cells expressing Parkin (iRFP positive cells; panels iii–v). Consistent with our other findings, CCCP treatment did not induce mitophagy in iRFP-negative cells lacking Parkin (panels vi–x, solid white arrow), but induced mitophagy in iRFP-positive cells expressing Parkin (panels viii–x). (**H**) FCM analysis was used to demonstrate the requirement of Parkin expression for CCCP-induced mitophagy. Cells stably expressing Mito-Rosella and transiently transfected with iRFP-T2A-Pakin were first gated according to negative vs. positive iRFP-fluorescence (*i.e.,* No Parkin vs. Parkin expression, respectively), and then analyzed separately to detect mitophagic cells (see Suppl. Fig. 5D), using the same method described for Fig. 3C,D.

The robust Rosella biosensor responses observed in single cell measurements suggested they could be adapted for flow cytometry (FCM)-based assays, which are capable of making rapid, highly-quantitative measurements. We measured effects of CCCP treatment in cells transfected with Rosella-LC3 or Mito-Rosella and Parkin, using standard FCM methods. In cells expressing Rosella-LC3, CCCP induced a BafA1-sensitive reduction in green fluorescence (green-shift) indicating induction of autophagy (detected in 34.3 ± 1.2%, 44.3 ± 0.6%, and 60.0 ± 1.9% of cells measured at 6, 12 or 24 hr, respectively; Fig 3A,C). In cells expressing Mito-Rosella with Parkin, CCCP also induced a BafA1-sensitive green-shift indicating mitophagy (detected in 25 ± 0.2%, 49.8 ± 1.8 and 59.4 ± 1.3% of cells measured at 6, 12, or 24 hr after treatment, respectively; *p* < 10^−4^ vs. cells treated with CCCP + BafA1 for each of the three time-points; Fig. 3B,D). These responses correlated with Western blot analysis of LC3 processing (Fig. 3D) and disappearance of mitochondrial markers (Fig. 3F).

### A spectrally distinct fluorescent population marker (iRFP) can be used in screens to assess regulators of autophagy/mitophagy

High-throughput screens utilizing DNA or RNA expression libraries frequently employ fluorescent proteins to monitor transfection/transduction efficiencies as a means of improving signal-to-noise ratio. Since standard FCM instruments measure several fluorescent channels simultaneously, we tested whether a third fluorescent marker could be used in conjunction with green/red Rosella biosensors to monitor responses selectively in transfected cells only. To test this, we transiently transfected Parkin coupled via a T2A sequence^39^ to near-infrared fluorescent protein^40^ (iRFP) in cells stably expressing Mito-Rosella (Fig. 3G). Transient transfection efficiencies in HeLa are ~60–70%, so that a significant number of cells go untransfected under these conditions. The FCM analysis was gated for iRFP fluorescence as the indicator of Parkin expression (Suppl. Fig. 5). Cells expressing Parkin (60% the total population) exhibited robust CCCP-induced mitophagy, whereas untransfected cells showed no mitophagy (Fig. 3H). Therefore, use of a spectrally distinct fluorescent marker in conjunction with Rosella enables robust analysis even under imperfect transfection/transduction conditions.

### Characteristics of Rosella-based flow cytometry parallel autophagy/mitophagy assay in HeLa cells

We performed additional studies to characterize the performance of the FCM assay for mitophagy. We determined the sensitivity of the assay for detecting CCCP-induced mitophagy at earlier time-points. As shown in Suppl. Fig. 3A, a statistically significant increase in mitophagy was detected at 2 hr after treatment with CCCP (7.4 ± 0.4% vs. 5.4 ± 0.3% for CCCP + BafA1 treatment, *p* < 0.01). We also compared the efficiency of Parkin translocation to mitochondria at 6 and 24 hr after treatment with 10 μM CCCP. As expected, there was extensive translocation of Parkin to mitochondria at 6 and 24 hr (70.1 ± 2.3% and 95.4 ± 1.7%, respectively). The percentage of cells displaying mitophagy at these time-points (Fig. 3D) is less than the percentage displaying Parkin translocation (Suppl. Fig. 3B), which agrees with prior observations that Parkin translocation to mitochondria occurs more rapidly and efficiently than mitochondrial clearance by mitophagy in CCCP-treated HeLa cells^32^. However, rates of mitophagy in the HeLa model can be increased by treatment with higher concentrations of CCCP, as a dose-response study showed 20 μM CCCP induced greater mitophagy than the 10 μM concentration used in our studies (Suppl. Fig. 3C). To determine whether the relative amount of Mito-Rosella expression alters measurements of rates of mitophagy, we studied cells co-transfected with a fixed amount of Parkin and varying amounts of Mito-Rosella. As shown in Suppl. Fig. 3D, there is no significant difference in the quantities of CCCP-induced mitophagy measured over a 3-fold range of transfected Mito-Rosella.

It has been reported that under certain conditions, CCCP may directly alter lysosomal function^41^. Since the FCM-based Rosella method relies on acidification of lysosomes to detect delivery of autophagic cargo, we tested whether CCCP treatment alters lysosome acidity in our model. As shown in Suppl. Fig. 4A,B, there were no changes in lysosome acidification induced by CCCP under the conditions of our experiments, as measured by Lysotracker red uptake. Since CCCP-induced, Parkin-dependent mitophagy relies on depolarization of mitochondria, we also tested the efficiency of CCCP in depolarizing mitochondria in our model. As shown in Suppl. Fig. 4C–D, CCCP induced substantial depolarization of mitochondria at 6, 12, and 24 hr after treatment, as measured by uptake of the membrane potentiometric dye TMRE. Interestingly, at 24 hr after treatment with CCCP, a partial recovery of mitochondrial membrane potential was evident (74.4% depolarization at 24 hr vs. 94.4% and 91.6% at 6 and 12 hr, respectively), which could potentially affect the maximal rates of mitophagy measured at 24 hr in this model.

**Figure 4:**
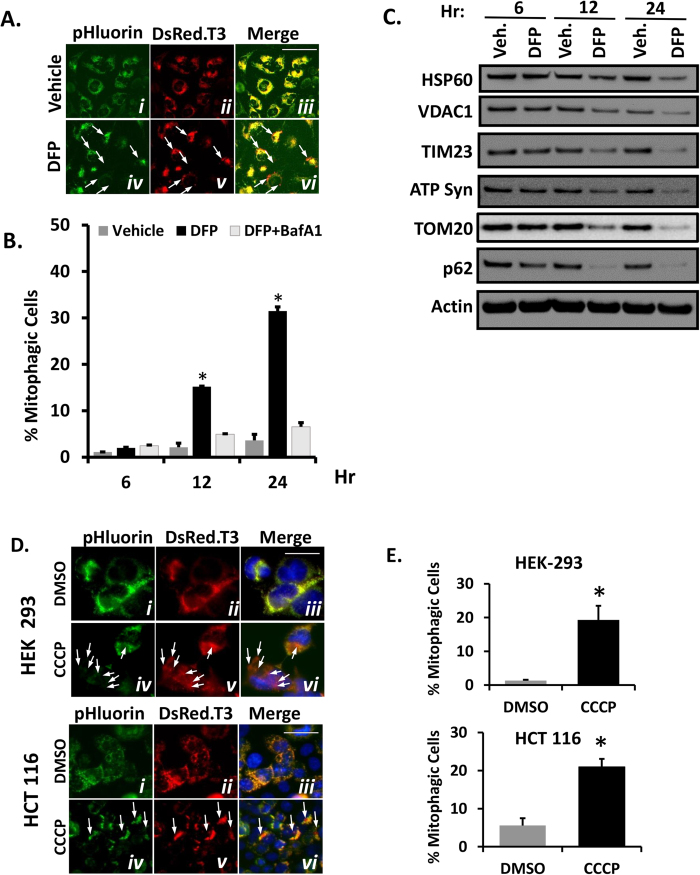
Quantification of CCCP induced mitophagy in HEK-293 and HCT-116 cells and deferiprone (DFP)-induced mitophagy in HeLa cells. (**A**) Representative micrographs of HeLa cells transfected with Mito-Rosella and then treated with 1mM of 3-Hydroxy-1,2-dimethyl-4(1H)-pyridone (deferiprone, DFP) or Vehicle (phosphate-buffered saline, PBS) for 24 hr. DFP treatment caused an accumulation of red-only Mito-Rosella labeled mitochondria (panel vi, solid arrows) while in vehicle treated cells there was complete co-localization between red/green signals (panel iii). Scale bar indicate a length of 50 μm. (**B**) Percentage of Mito-Rosella expressing cells showing increased mitophagy after 1 mM DFP treatment at indicated time-points as quantified by mito-Rosella FCM assay. Bars represent mean of 3 biological replicates ± SEM, N = 10,000 cells analyzed per replicate; *p < 0.001 vs DFP + BafA1 (**C**) Western blots of markers of mitochondrial content–HSP60, ATP synthase, VDAC1, TIM23 and TOM20 (top four panels) and p62 (autophagy marker) and actin (gel loading control) in cell treated with DFP or Vehicle (PBS) control for the indicated time-points. (**D**) Representative micrographs of HEK-293 cells transfected with Mito-Rosella alone (top panels) and HCT-116 cells (bottom panels) transfected with Mito-Rosella plus HA-Parkin; cells were treated with CCCP (10 μM) or vehicle as indicated; treatment durations were 24 hr for HEK-293 cells or 6 hr for HCT-116 cells. CCCP treatment induced accumulation of red-only Mito-Rosella indicating mitophagy (white arrows). Scale bars indicate a length of 25 μm. (**E**) Percentage of Mito-Rosella expressing HEK-293 and HCT-116 cells showing increased mitophagy after CCCP treatment as quantified by mito-Rosella assay.

To determine the utility of the Mito-Rosella-based assay for measuring mitophagy induced by other stimuli, we tested its ability to quantify mitophagy in HeLa cells treated with the iron chelator, 3-Hydroxy-1,2-dimethyl-4(1H)-pyridone (deferiprone, or DFP), which is reported to induce mitophagy independently of Parkin^42^. As shown in Fig. 4A–C, treatment of HeLa cells expressing Mito-Rosella (without Parkin) with DFP (1 mM) induced mitophagy at 12 and 24 hr after treatment (Fig. 4A,B). The induction of mitophagy by DFP correlated with Western blot analysis showing a decrease in mitochondrial markers (Fig. 4C). We also tested the ability of the Rosella-based mitophagy assay to quantify mitophagy in other cell types beside HeLa. As shown in Fig. 4D,E, the assay readily detects mitophagy in CCCP-treated HEK-293 kidney epithelial cells, which are known to express endogenous Parkin^31^,^32^,^43^,^44^, as well as in CCCP treated HCT-116 colorectal carcinoma cells co-transfected with Parkin. In addition, the Mito-Rosella-based assay exhibits similar capacity to measure mitophagy in HepG2 hepatocarcinoma cells, H9C2 cardiomyocytes, and bovine aortic endothelial cells (*data not shown*).

## Discussion

Degradation of mitochondria by macroautophagy, also known as ‘mitophagy’, is important for mitochondrial function and has been implicated in human disease processes. Mitophagy requires autophagy, and so autophagy and mitophagy must be assessed in parallel when considering regulation of mitophagy. Here we report a novel, flow cytometry (FCM)-based assay for measuring autophagy and mitophagy in parallel using a single fluorescent biosensor, Rosella, targeted to different cellular compartments. We demonstrate the performance of this assay in measuring Parkin-mediated mitophagy in CCCP-treated HeLa, a well-characterized model of selective mitophagy, in HEK-293 and HCT-116 cells treated with CCCP, and in Parkin-independent mitophagy in cells treated with DFP. Measuring 10,000 cells requires 6 seconds of analysis time (<10 min for 96 separate conditions). Therefore, this parallel assay for autophagy/mitophagy has the capacity to make hundreds to thousands of measurements in minutes to hours. Moreover, by combining these methods with iRFP as a marker of genetic manipulation (as shown in Fig. 3G,H), the assay should be capable of screening large libraries of cDNA, RNAi, TALEN, or Crispr-Cas9 sgRNA to identify regulators of selective mitophagy.

Our findings indicate that LC3-Rosella and Mito-Rosella can be employed in parallel to measure both generalized macroautophagy and selective mitophagy; and importantly, the two biosensors used together can readily detect dissociation between autophagy and mitophagy when it occurs (*e.g.,* in HeLa cells treated with CCCP but not expressing Parkin). Other fluorescent markers have been used for monitoring delivery of autophagic cargo lysosomes, including tandem mCherry-eGFP^45^,^46^, mRFP-GFP^47^, and RFP-eGFP^48^ constructs, and the pH-sensitive Keima^49^ fluorescent protein; however, this is the first demonstration of an FCM-based high-throughput method for measuring autophagy and mitophagy in parallel in mammalian cells.

Monitoring of mitochondrial content by means of Western blotting of mitochondrial protein markers, uptake of mitochondria-specific dyes, or measurement of mitochondrial DNA provides information about the cellular content of mitochondria at any given point in time. However, because the net content of mitochondria reflects simultaneous mitochondrial biogenesis and mitochondrial turnover, mediated in part by mitophagy, these methods may underestimate mitophagy when there is substantial mitochondrial biogenesis. Because measurement of mitochondrial flux to the lysosome by the Mito-Rosella method is a more direct measurement of mitophagy (*i.e.,* trafficking via autophagy and terminal delivery of mitochondria to the functional lysosome), there is less potential for it to be affected by mitochondrial biogenesis than surrogate measurements of mitophagy based on mitochondrial content.

Moreover, several of the mitochondrial dyes used to quantify mitochondria–including those described as ‘non-potentiometric’^50^ can be affected by conditions that alter membrane potential^51^,^52^ and should be used with caution. For example, staining of mitochondria with 10-N-nonyl acridine orange (NaO), a dye that interacts specifically with cardiolipin on the mitochondrial membrane, is highly dependent on mitochondrial energy state^53^. Moreover, recent reports that externalization of cardiolipin to the outer mitochondrial membrane plays a role in initiation of mitophagy^24^ raises questions about how this process might influence measurements of mitochondrial mass using NaO. In addition, a recent report by Padman *et al.*^41^ showed redistribution of mitochondrial dyes (DiOC6, TMRM, MTR, and MTG) from mitochondria to lysosomes after CCCP treatment. Another aspect of non-potentiometric mitochondrial dyes is that they typically have affinity for some specific chemical group on/in mitochondria, and these dyes can therefore potentially stain mitochondria even after delivery to lysosomes via mitophagy, which could result in overestimation of intact mitochondrial mass.

Because Rosella monitors terminal delivery of cargo to lysosomes, the assay cannot provide information about lysosome-independent mechanisms for mitochondrial degradation^54^, or budding of mitochondrial-derived vesicles (MDVs) that may traffic to other compartments^55^. A potential limitation of this method is that Rosella biosensors could potentially detect lysosome-independent acidification of cargos; conversely, they could underestimate delivery of cargos when lysosomal pH is elevated by other factors^41^. Therefore, pilot studies to assess intracellular localization of different biosensor-tagged cargos by fluorescence microscopy and sensitivity to BafA1 treatment are advisable when applying these methods to new conditions or when attaching Rosella to different cargos.

Taken together, these findings indicate Rosella biosensor measurements are amenable to use in high-throughput screening assays designed to identify chemical or genetic factors that regulate autophagy or mitophagy in mammalian cells, and can readily detect selective regulation of mitophagy as a dissociation between the two processes when it occurs. We are currently applying the Rosella-based autophagy/mitophagy assays to screen shRNAi and cDNA libraries in an effort to identify new regulators in mammalian cells, and we are producing transgenic mice expressing Rosella-LC3 and Mito-Rosella biosensors for measuring autophagic and mitophagic fluxes to lysosomes in different tissues *in vivo*.

## Materials and Methods

### Cell culture

HeLa, HEK-293 and HCT-116 cells were from ATCC (American Type Culture Collection), and cultured in DMEM for HeLa and HEK-293 (Life Technologies) and McCoy's 5a Medium Modified (Life Technologies) for HTC-116 and supplemented with 10% heat-inactivated fetal bovine serum (Atlanta Biologicals), penicillin, and streptomycin (Life Technologies), and maintained in a 5% CO_2_, 37 °C humidified incubator. Media was changed every third day, and culture passage number was maintained under fifteen.

### Expression plasmids

The Rosella biosensor was described in previous publications^35^,^36^. The full-length open reading frame (ORF) of human microtubule-associated protein 1A/1B-light chain 3 (Map1lc3b) was joined in-frame to the 3′ end of the Rosella ORF (coding the C-terminus) in the pDest expression vector, generating the Rosella-LC3 biosensor (Fig. 1A), for use in detecting delivery of autophagasomes to lysosomes (*i.e.,* generalized macroautophagy). The ATP synthase subunit gamma (Atp5c) full-length ORF minus the terminal stop codon was joined in-frame to 5′ of the Rosella ORF (end (coding the N-terminus) in the pCIneo expression plasmid (Clontech), generating the Mito-Rosella biosensor (Fig. 1A) for detecting delivery of mitochondria to lysosomes (*i.e.*, mitophagy). pRK5-HA-Parkin (Addgene plasmid 17613) was a gift from Dr. Ted Dawson (Johns Hopkins University), pIRFP (Addgene plasmid 31857; based on vector backbone pEGFP-C1) was a gift from Dr. Vladislav Verkhusha (Yeshiva University). iRFP-T2A-Parkin vector was generated from IRFP-T2A-TREX2 in pExodus CMV vector (Addgene plasmid #50419), a gift from Dr. Andrew Scharenberg (Seattle’s Children Hospital), by substituting TREX2 with Parkin. pmTurquoise2-Mito (Addgene plasmid 36208) was a gift from Dr. Dorus Gadella (University of Amsterdam) and mCherry-Parkin (Addgene plasmid 23956) was a gift from Dr. Richard Youle (NINDS). HeLa cells with stable expression of Rosella-LC3 or Mito-Rosella were generated by cloning the biosensor ORFs into a PiggyBac transposon transfer vector (System Biosciences, product #B510B-1), which was then co-transfected with the Super PiggyBac Transposase expression vector (System Biosciences, product #PB210PA-1); stable cell lines were developed by repeated passaging in the presence of puromycin (2 μg/mL, InvivoGen) for 3 weeks, followed by sterile flow cytometry-automated cell sorting to isolate cells with increased green and red channel fluorescence. After sorting, stable lines were cultured continuously in media containing Puromycin (2 μg/mL).

### Microscopy and flow cytometry (FCM)

Figure 1E cells were plated on 60 mm glass bottom poly-D-lysine coated plates, transfected with Rosella-LC3 or Mito-Rosella with Parkin. 24 hr post transfection cells were treated as indicated and live images were obtained with Nikon Eclipse Ti-E widefield inverted microscope equipped with 405 nm diode laser, 488 nm Argon gas and 543 nm Helium-neon gas lasers and DAPI (450–465 nm) FITC (505–535 nm) and TRITC (580–620) filter sets using 100X oil immersion objective. **For all other images:** Cells were grown on 6 or 12 well plates and images of unfixed, live cells were obtained using an EVOS-fl inverted LED fluorescence microscope. pHluorin emission (510 ± 42 nm), DsRed.T3 emission (593 ± 40 nm) and iRFP emission (692 ± 40 nm) were measured after excitation with (470 ± 22 nm), (531 ± 40 nm) and (628 ± 40 nm) LED illuminations respectively. ImageJ software were used for quantitative analysis. Individual cells in micrographs were scored as being positive or negative for autophagy or mitophagy, based on the presence/absence of visible accumulations of red biosensor fluorescence in the central lysosomal compartments with distinct “red only” fluorescence. For FCM, cells were grown on 12 well plates until reaching 90% confluency and treated with CCCP (10 μM) and/or bafilomycin A (100 nM,) for indicated durations. Cells were harvested for analysis by washing twice with PBS, trypsinizing with TrypLE (Life Technologies), and transferring to 96 well plate wells containing 0.3 mL PBS. Cells were analyzed using a BD FACSCanto analyzer on high throughput mode. pHluorin emission (530 ± 30 nm), DsRed.T3 emission (585 ± 15 nm) and iRFP emission (780 ± 60 nm) were measured after excitation with 488, 561 and 640 nm lasers, respectively. 5,000 to 10,000 live cells were analyzed per condition, with fluorescent detection in green and red channels, and when required for detecting iRFP, the near-infrared (Cy7) channel. Increased autophagy or mitophagy were determined for individual cells by detecting decreased green vs. red fluorescence, based on gating determined by the green and red fluorescence of vehicle (DMSO)-treated control cells. To eliminate potential confounding effects of lysosome-independent changes in mitochondrial pH, Bafilomycin A1-treated cells were used as a control condition under situations where potential acidification of media or cytoplasm might occur (*e.g.,* prolonged CCCP treatment in Fig. 3H).

### Antibodies and chemicals

Primary antibodies used in this study: rabbit anti-LC3A/B, rabbit anti-Parkin rabbit anti-HSP60 (1:1000, Cell Signaling, products #4108S, #4211S and #12165S , respectively); mouse anti-TIM23 (1:1000, BD Biosciences, product #611222); mouse anti-VDAC1, mouse anti-TOM20, and mouse anti-p62 (Santa Cruz, 1: 500, sc-58649, sc-17764, and sc-28359, respectively); rabbit anti-ATP synthase gamma (1:1000, GeneTex, product #GTX-114275), mouse anti-Actin (1:3000, Genescript, product #A00702) and rabbit anti-GFP (Genescript, product #A01388-40). Secondary antibodies were: HPR conjugated goat anti-rabbit and goat anti-mouse (1:2500, products #170-6515 and #172-1011, respectively). CCCP and DMSO were obtained from Sigma. Bafilomycin A1 was obtained from LC Laboratories. Mitotracker Deep Red FM, Lysotracker Red DND 99, and TMRE, and Hoechst 33342 were obtained from Life Technologies. 3-Hydroxy-1,2-dimethyl-4(1H)-pyridone was from Sigma.

### Western blot analysis

Cells were washed twice in PBS and lysed with ice-cold RIPA buffer (Santa-Cruz) containing HALT protease/phosphatase cocktail (Pierce/Thermo Scientific). Lysates were incubated on ice for 30 min with gentle, constant agitation. Lysates were centrifuged at 12,000 × g for 20 min at 4 °C to pellet nuclei and other insoluble material. Lysates were treated with Novex sample buffer and heated 10 min at 90 °C. SDS-PAGE was performed on 4–12% Tris-Bis gels (Life Technologies) with MES-based running buffer containing anti-oxidant additive, according to the manufacturer's instructions. Gels were transferred to nitrocellulose (iBlot system, Life technologies). Western blot membranes were blocked with 5% BSA in TBS-T buffer for 1 hour at room temperature, followed by incubation with 5% BSA in TBS-T containing primary antibodies at the indicated concentrations (see Methods, above) overnight at 4 °C with gentle rocking; in the morning, blots were washed in TBS-T and incubated 1 hr at room temperature in 5% BSA in TBS-T containing HRP-conjugated secondary antibodies (1:2500), washed again in TBS-T, and incubated in luminescent peroxidase substrate (ThermoFisher). Imaging of Western blot band luminescence was performed with a GeneGnome imager (Syngene); and band intensities were quantified using Gene Snap software (Syngene).

## Additional Information

**How to cite this article**: Sargsyan, A. *et al.* Rapid parallel measurements of macroautophagy and mitophagy in mammalian cells using a single fluorescent biosensor. *Sci. Rep.* **5**, 12397; doi: 10.1038/srep12397 (2015).

## Supplementary Material

Supplementary Information
