# Supplementary material for: Rapid parallel measurements of macroautophagy and mitophagy in mammalian cells using a single fluorescent biosensor
Source: Sci Rep. 2015 Jul 28;5:12397. doi: 10.1038/srep12397 (PMC4517063; doi:10.1038/srep12397)

Supplemental Figures

Rapid parallel measurements of macroautophagy and mitophagy in mammalian cells using a single fluorescent biosensor

Sargsyan A, Cai J, Fandino LB, Labasky ME, Forostyan T, Colosimo LK, Thompson SJ, Graham TE

SUPPLEMENTAL FIGURE LEGENDS

Suppl. Figure 1. Localization of untagged Rosella. HeLa cells expressing untagged Rosella were treated 12 hr with CCCP (10 μM), with or without BafA1 (100 nM) as indicated. Scale bar represents 50 μm distance.

Suppl. Figure 2. Analysis of effects of Rosella-LC3 and Mito-Rosella on dynamics of autophagy and mitophagy. (A) Expression of Rosella-LC3 or Mito-Rosella do not effect dynamics of mitochondrial marker Hsp60, ATP synthase, TOM20, and VDAC1 degradation in CCCP-treated (10 μM) HeLa cells. Detection of Rosella constructs with anti-LC3 antibody (Rosella-LC3 only) or anti-GFP antibody (Rosella-LC3 and Mito-Rosella) is shown; two exposures are shown for the anti-GFP blot because the antibody recognizes Mito-Rosella less avidly than LC3-Rosella. **(B)** Lack of effect of Rosella-LC3 or Mito-Rosella on dynamics of autophagy in CCCP-treated HeLa cells, as measured by processing of endogenous LC3. Bafilomycin-A1 (BafA1, 100 nM) treatment was employed as indicated to detect the flux of LC3 processing.

Suppl. Figure 3. Variables related to Rosella biosensor assay performance in CCCP-treated HeLa cells. (A) Time course of mitophagy measured by mito-Rosella in HeLa cells expressing Parkin and treated with CCCP (10 μM); (**B)** Parkin translocation to mitochondria in HeLa cells treated with CCCP (10 μM), as imaged at 6 or 24 hr; bar graph (right) shows quantification of percentage of cells with Parkin translocation; bars represent median ± SEM, n=120-200 cells analyzed per condition. **(C)** CCCP dose-response of mitophagy measured by mito-Rosella method (*p<0.01 vs DMSO treated, #p<0.01 vs lower dose of CCCP). **(D)** Lack of effect of varying levels of mito-Rosella expression on mitophagy measurements; the inset table indicates relationship between transfected plasmid quantities and mito-Rosella expression, as measured by dsRed.T3 fluorescence intensity per cell.

Suppl. Figure 4. **Lysosome status and mitochondrial membrane potential in CCCP-treated HeLa cells. (A)** HeLa cells were treated with vehicle (DMSO) or CCCP (10 μM) and then stained with Hoechst nuclear dye (blue) and Lysotracker Red dye (red). **(B)** Lysotracker fluorescence was measured by FACS analysis after treatment for the indicated times; bars represent the mean ± SEM intensity per cell of 3 individual biological replicates per condition, n=5,000 individual cells measured per replicate. There is no inhibitory effect of CCCP on lysosome content or Bafilomycin A1 (BafA1)-sensitive (*i.e.*, pH-dependent) uptake of Lysotracker dye. **(C)** HeLa cells expressing mitochondrial-targeted pmTurquoise2 (Trq2-Mito) were treated with vehicle (DMSO) or CCCP (10 μM) for the indicated times, and then stained with TMRE, a mitochondrial membrane potential (MMP)-dependent dye. As observed for "red-only" mito-Rosella, CCCP treatment causes Trq2-Mito to redistribute to the perinuclear central lysosomal region. **(D)** TMRE:Trq2-Mito

fluorescence ratio per cell was measured by FACS analysis after treatment for the indicated times; bars represent the mean ± SEM of the TMRE:Trq2-Mito fluorescence ratio for 3 biological replicates per condition, n=5,000 individual cells measured per replicate. TMRE:Trq2-mito fluorescence is suppressed >90% after 6 or 12 hrs of CCCP-treatment, indicating near-total loss of MMP; after prolonged 24 hr CCCP treatment, there is partial recovery of MMP (**p*<0.01 for 24 hr vs. 6 or 12 hr measurements).

Suppl. Figure 5. Analysis of CCCP induced autophagy and mitophagy in HeLa cells expressing Rosella LC3 or mito-Rosella with Parkin by means of standard Flow Cytometry (FCM). (A) Hela cells expressing Rosella LC3 or Mito-Rosella and Parkin (n=10,000 each, were analyzed by standard two channel (green/red) FCM. Cells with increased autophagy (Rosella-LC3, upper panel) after treatment with CCCP (10 µM, 24hr) were detected on the basis of changes in green (pHluorin) vs. red (DsRed.T3) fluorescence, relative to vehicle (DMSO)-treated cells. Both CCCP-induced autophagy and mitophagy were inhibited by BafA1, indicating the responses require lysosomal acidification. **(B)** Time course analysis of induction of FCM-measured autophagy in HeLa cells expressing Rosella-LC3 without Parkin; **p*<0.001 vs CCCP+BafA1 for indicated time-points; #*p*<0.05 for 24 hr vehicle (DMSO) treated cells vs 6 or 12 hr vehicle (DMSO) treated cells. **(C)** Mitochondrial proteins (VDAC1, TIM23, ATP synthase and HSP60) and p62 were measured by Western blotting in lysates of HeLa cells after treatment with CCCP (10µM) or vehicle (DMSO) for the indicated times. **(D)** HeLa cells were transiently transfected with a CMV promoter-driven bicistronic expression plasmid containing the ORF of near-infrared fluorescent protein (iRFP) fused to the Parkin ORF via a T2A ribosomal ‘’skip’’ region; presence of the T2A region causes

a precise interruption in translation, resulting in equal expression of iRFP and Parkin as separate polypeptides. Separate populations of untransfected and Parkin transfected cells were distinguished on the basis of low or high levels of iRFP fluorescence (left panel). Gating for iRFP fluorescence enabled simultaneous population specific FCM measurement of mito-Rosella fluorescence in the green and red channels, making it possible to separately determine percentages of cells showing increased mitophagy in each population (untransfected vs Parkin-transfected) under the exact same treatment conditions (upper and lower right panels).

Suppl. Figure 1

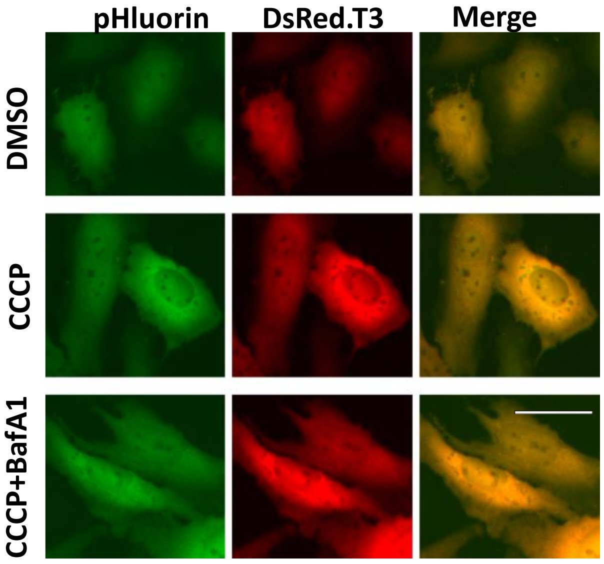

Suppl. Figure 2

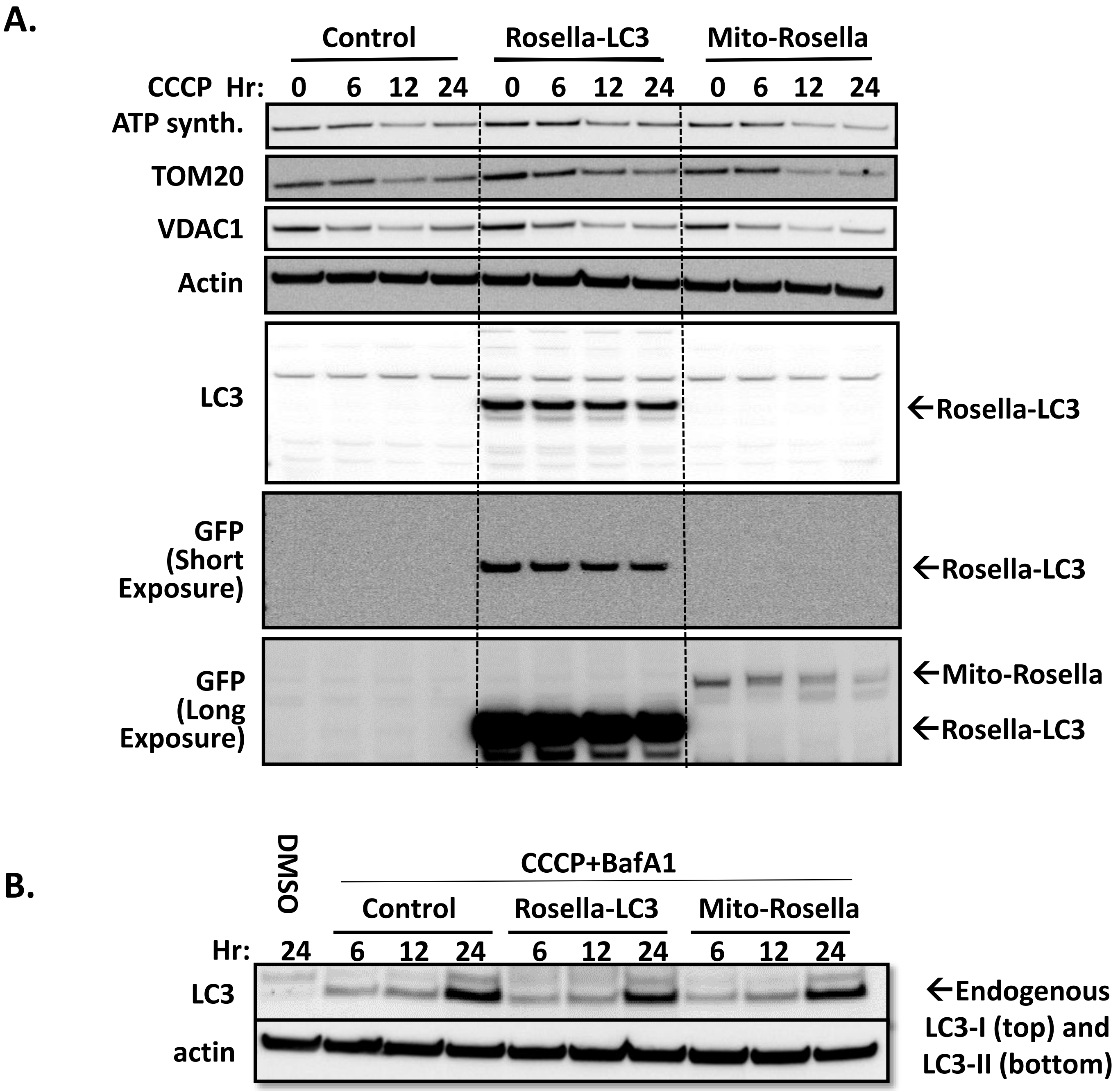

Suppl. Figure 3

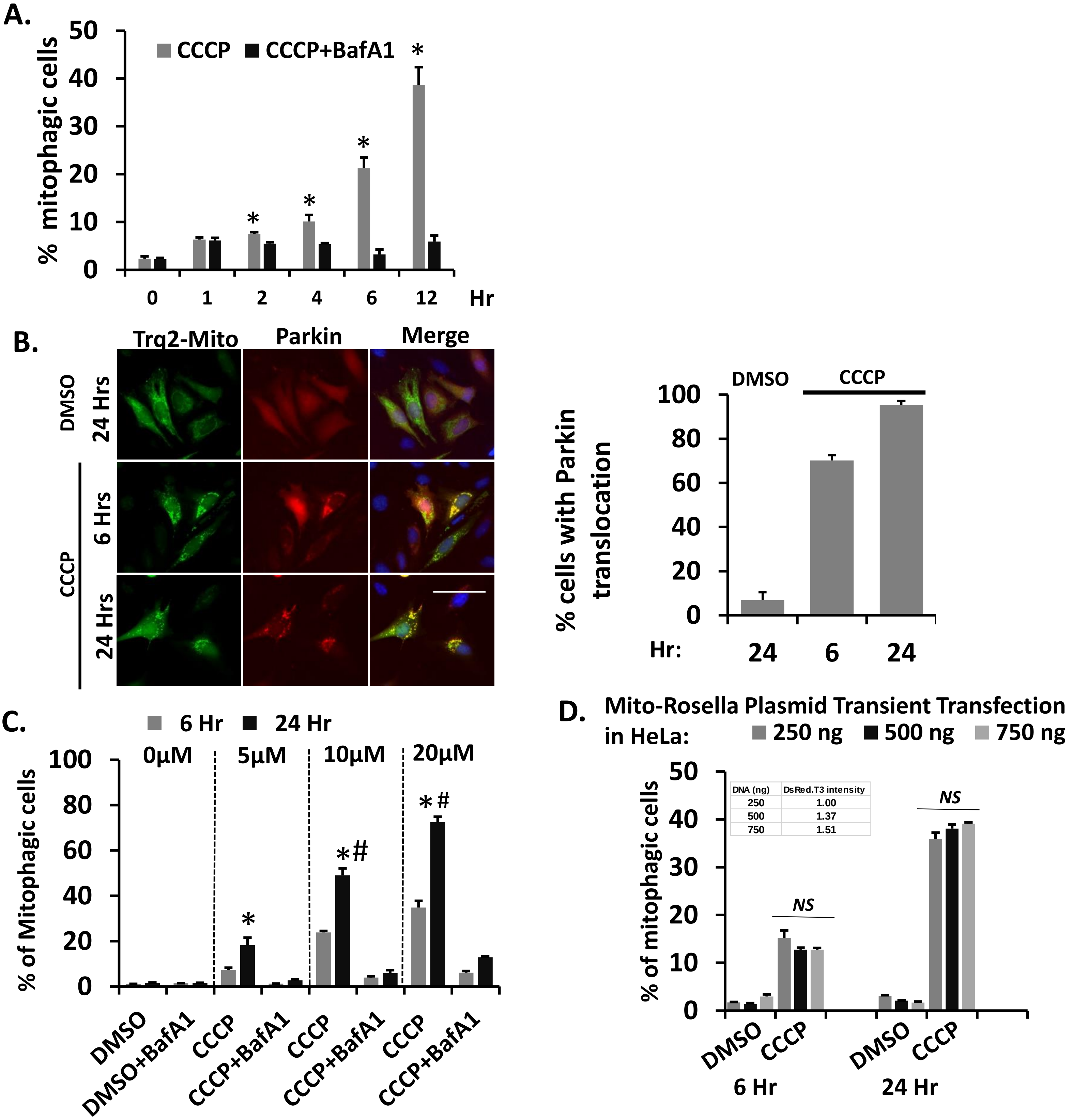

Suppl. Figure 4

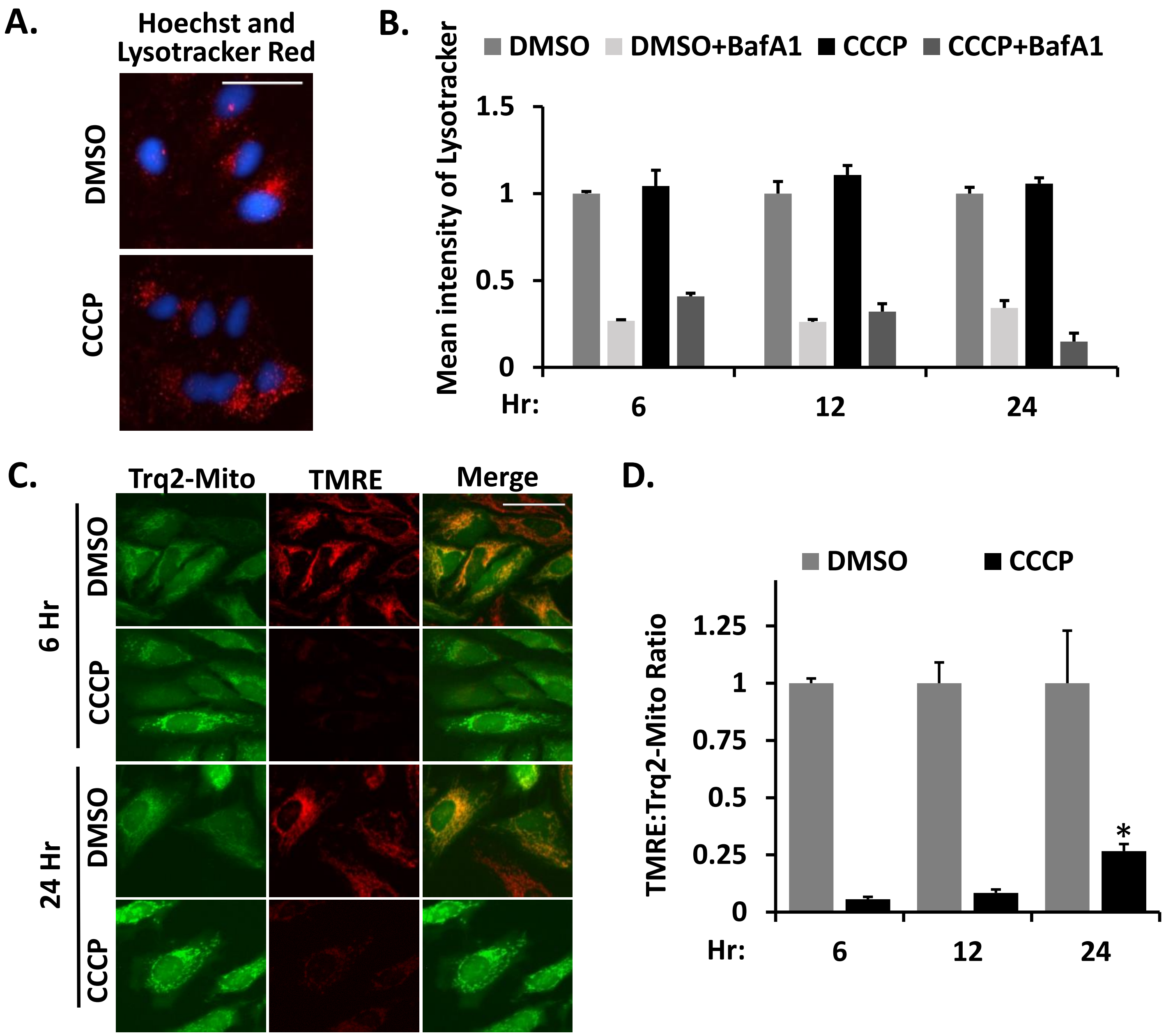

Suppl. Figure 5

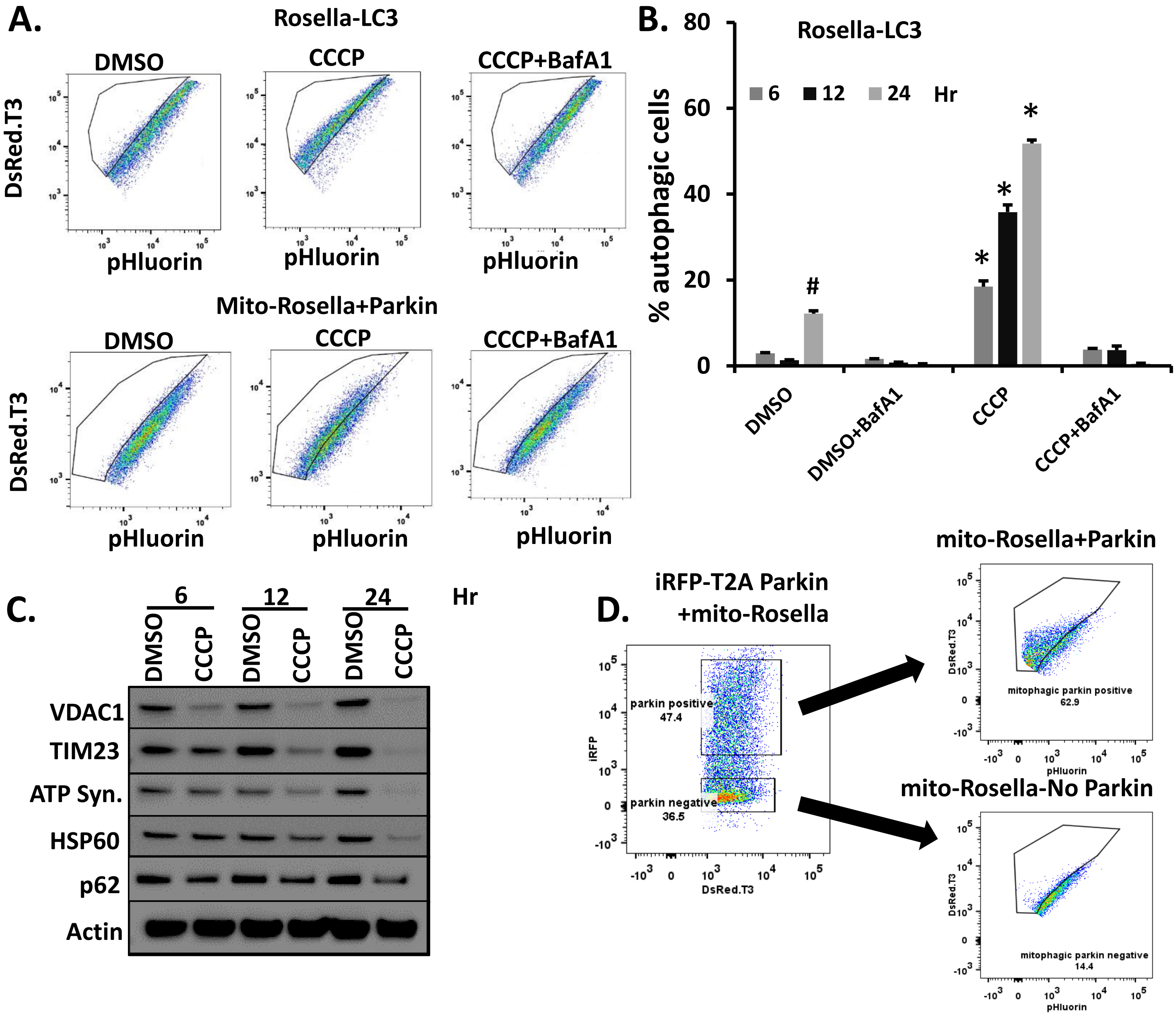

Supplement: Supplementary Information [file srep12397-s1.pdf]
